# Optofluidic paper-based analytical device for discriminative detection of organic substances via digital color coding

**DOI:** 10.1038/s41378-024-00865-4

**Published:** 2025-01-16

**Authors:** Jinsol Choi, Chi Yeung Oh, Gong Qian, Tae Soup Shim, Heon-Ho Jeong

**Affiliations:** 1 Department of Chemical and Biomolecular Engineering, Chonnam National University, 50 Daehak-ro, Yeosu-si, Jeollanam-do 59626 Republic of Korea; 2 Department of Energy Systems Research, Ajou University, 206 World cup-ro, Suwon-si, Gyeonggi-do 16499 Republic of Korea; 3 Department of Chemical Engineering, Ajou University, 206 World cup-ro, Suwon-si, Gyeonggi-do 16499 Republic of Korea

**Keywords:** Structural properties, Optical sensors

## Abstract

Developing a portable yet affordable method for the discrimination of chemical substances with good sensitivity and selectivity is essential for on-site visual detection of unknown substances. Herein, we propose an optofluidic paper-based analytical device (PAD) that consists of a macromolecule-driven flow (MDF) gate and photonic crystal (PhC) coding units, enabling portable and scalable detection and discrimination of various organic chemical, mimicking the olfactory system. The MDF gate is designed for precise flow control of liquid analytes, which depends on intermolecular interactions between the polymer at the MDF gate and the liquid analytes. Subsequently, the PhC coding unit allows for visualizing the result obtained from the MDF gate and generating differential optical patterns. We fabricate an optofluidic PAD by integrating two coding units into a three-dimensional (3D) microfluidic paper within a 3D-printed cartridge. The optofluidic PADs clearly distinguish 11 organic chemicals with digital readout of pattern recognition from colorimetric signals. We believe that our optofluidic coding strategy mimicking the olfactory system opens up a wide range of potential applications in colorimetric monitoring of chemicals observed in environment.

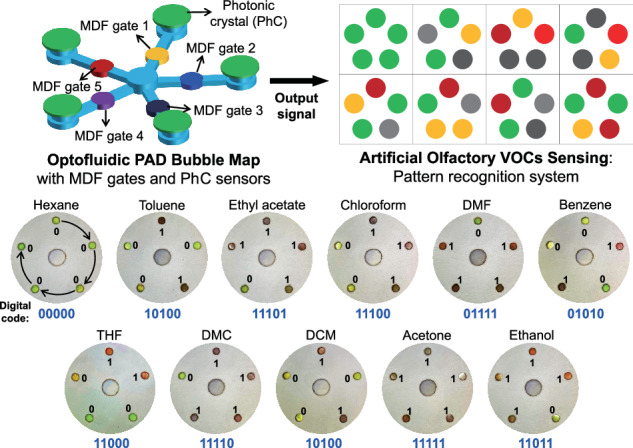

## Introduction

Most chemical substances used in industrial fields, especially volatile organic compounds (VOCs), are well-known to cause serious health risks to humans, such as carcinogenesis as well as immune, kidney, and liver damage, so the identification of a wide range of chemical substances is of great interest in both industry and everyday life^1^. All chemical substances have “fingerprints”—differences in size, shape, polarity, chirality, and reactivity. These differences can provide specific starting points to develop and improve the performance of chemical detection. The main approach to chemical sensing is transforming information on physical or chemical changes from interactions between the analyte and the sensing unit into a distinguishable output signal. For example, gas and liquid chromatography are highly sensitive, accurate, and versatile techniques that use interactions between analytes in the mobile phase and resins in the stationary phase resulting in varying retention times. However, these methods are costly and demand considerable time, space, and operation skill level.

One of the most reliable and sophisticated chemical detection found in nature is the human olfactory system, which can discriminate over a trillion odors by using hundreds of olfactory receptors^2^. However, this natural system struggles to detect colorless and odorless compounds due to the absence of specific receptors. Inspired by nature, artificial nose and tongue sensors have been exploited to detect various organic compounds that transmit signals through different coding mechanisms (combinatorial coding for olfaction and labeled line coding for taste)^3^. This is achieved by using various detection probes and transduction techniques that enable simple and sensitive measurements based on, for instance, chemoresistive or electrochemical sensors, field effect transistors, and optical sensors^4^.

Among detection technologies, the optical sensor array, generally called an optoelectronic nose, has emerged as a versatile approach to identify VOCs by pattern recognition of signal change of the arrayed sensing probes^5,6^. This multiplex sensing platform mimics the olfactory system, which is advantageous for differentiating extremely diverse types of chemical substances. The combinatorial information on signals from sensor arrays facilitates the detection of various chemicals by providing unique optical patterns for each target sample. A colorimetric sensor array offers naked eye detection based on changes in colors and enables rapid and facile on-site analysis of VOCs using portable devices, such as smartphones or tablets, instead of bulky equipment^7^. Typically, a colorimetric sensor uses dyes (e.g., Bronsted, solvatochromic, and redox dyes) as the sensory material that changes its optical properties by specific physicochemical interaction with the target^8^. However, for an optoelectronic sensing probe, dyes must be designed for each target, and long-term stability issues due to the degradation need to be improved. Recently, specific catalytic activity has also been utilized by using metallic nanomaterials which showed a potential for molecular-specific colorimetric sensors^9^. Another type of colorimetric sensory material is photonic crystal (PhC), which exhibits structural colors, as its regular crystal lattice determines the photonic bandgap. Thus, the color is semi-permanent, as long as the structure is retained. In addition, the color can be easily tuned by either expansion or constriction of the structure by environmental cues. Therefore, structural color materials have excellent long-term stability and are universally applied to various targets^10^.

An optofluidic device is a promising sensor platform that provides immediate sampling and detection of target fluid analytes. The best candidate as an optofluidic sensing platform is a microfluidic paper-based analytical device (PAD) that enables an efficient and low-cost assay owing to miniaturization, portability, point-of-care detection, no need for external pumps, and minimum reagent consumption^11,12^. Microfluidic PADs can be fabricated by selectively forming a hydrophobic barrier to induce and control aqueous fluid flow via capillary action in the hydrophilic paper channel. The fluid flow control in PADs is an important function for sensing capabilities with multiplex and multistep reactions^13^. Previous works report several methods for controlling fluid flow in PADs, such as designing certain channel geometry, valve integration, and material-based control of paper permeability^14–16^. In addition, the integration of colorimetric sensing probes in μPADs, such as inorganic nanoparticles, organic dyes, PhCs, and metal–organic frameworks, have been proposed to design an artificial olfactory sensing system via array-based multiplex assay for VOC sensing^17–20^. However, these approaches use a single sensing probe and thus are limited in scalability and versatility for detecting highly diverse chemical substances.

In this paper, we propose a paper-based optofluidic coding platform for discriminating a broad range of chemical substances through interpreting the differential pattern recognition of structural-color-derived optical signals. We accomplish the differential pattern recognition by using an optofluidic PAD comprising two types of coding units: macromolecule-driven flow (MDF) gate and PhC film. By exploiting the unique interactions between the solvents and the polymers, the optofluidic PAD controls the flow of liquid analytes in the MDF gate and induces changes in optical signals from the PhC film, enabling precise solvent discrimination. For more complex pattern recognition, the optofluidic PAD is designed to have a bubble-map array format with five MDF gates and five PhC sensors. We demonstrate that 11 organic chemicals are clearly distinguished with the digital readout of colorimetric signals from the bubble-map of the optofluidic PAD.

## Results and discussion

**Fig. 1:**
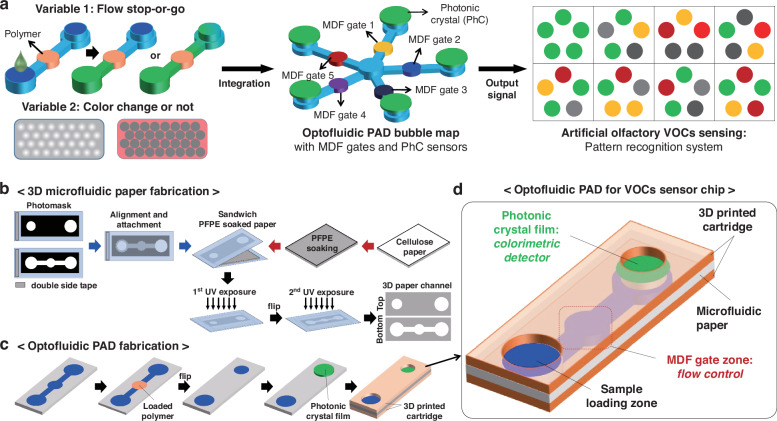
Schematic of the optofluidic coding workflow and fabrication of the optofluidic PAD comprising MDF gates and PhC. **a** Two variables—fluid flow control and structural color change—are considered to distinguish more chemical substances by varying types of MDF gate and structural color change of the PhC. These two coding units are integrated into a microfluidic paper of a bubble-map array format. After loading liquid analytes, the PhC coding unit generates a differential pattern of colorimetric change by chemical types. This output signal can be converted into a digital code, working as artificial olfactory coding. **b**–**d** Fabrication of the optofluidic PAD. **b** Two photomasks of different designs are aligned using double-sided tape. The paper soaked with a PFPE-DMA photocurable resin is placed between the photomasks. After two-sided exposure to UV light, a monolithic 3D microfluidic paper is formed. **c** A polymer is loaded to the center zone of the bottom channel, and the PhC film is placed on the top channel. They are then assembled using a 3D-printed cartridge. **d** Schematic of the resulting optofluidic PAD containing MDF gates and PhC film

The comprehensive coding strategy for discriminative detection of chemical substance is summarized in Fig. 1a. To construct a delicate pattern recognition system via optofluidic PAD, we introduce two types of coding units (left image in Fig. 1a). The primary coding units, so-called MDF gates, are aimed at determining the stop-or-go flow of liquid analyte in the middle zone of the channel. The MDF gates are prepared by infiltrating a polymer solution into the paper channels followed by drying to effectively fill the polymeric materials in the cellulose fibril network of the paper. This is designed to preemptively discriminate chemical substances based on the interaction between the mobile analytes and the stationary polymer. The secondary coding unit, a PhC film composed of non-close-packed colloidal crystal structure, is introduced to visualize the presence of chemical substances by structural colors. The polymer-based PhC film enables the naked eye readout of optical signals through changes in structural color when the solvent arrives. In addition, the difference in the structural color change in different solvents passing through the MDF gate allows for their precise discrimination. The combination of the two coding units generates complex color pattern maps, indicating the discrimination of various chemical substances, thus mimicking natural olfactory system. When introducing multiple MDF gates composed of different polymers into the bubble-map array, each solvent may show different flow patterns downstream (middle image in Fig. 1a). Those analytes that pass through the MDF gates reach the detection zone, causing a change in the reflection color of the PhC. The readout signal from the PhC coding unit array can be used to display a digital pattern for distinguishing chemical substances (right image in Fig. 1a). The fabrication procedure of the integrated optofluidic PAD with MDF gate and PhC coding units for reliable discrimination of chemical substances coding is shown in Fig. 1b, c. Firstly, we fabricate a monolithic microfluidic paper by double-sided photolithography using perfluoropolyether dimethacrylate (PFPE-DMA) (Fig. 1b). Photocurable liquid PFPE-DMA is used as the resin to form hydrophobic barriers with excellent solvent compatibility, which is suitable for fabricating flow channels for organic chemicals^21,22^. By using double-sided irradiation of UV light, we selectively form the hydrophilic paper channel region both on the top surface with two circular loading/detecting zones and the bottom surface with a hemi open channel (schematic illustrations and optical images in Fig. S1a). The 3D channel enables guiding the fluid in vertical and lateral directions, reduces sample evaporation, and provides accurate assay from uniform signals in the detection zone^23,24^. From the scanning electron microscopy (SEM) image, the boundary between the hydrophobic PFPE and the hydrophilic paper channel is clearly observed, and the morphology of the flow channel region reveals no difference compared to bare paper, indicating complete removal of uncured liquid PFPE-DMA (Fig. S1b). The MDF gates and PhC films are integrated into the microfluidic paper and then assembled with a 3D-printed cartridge (Fig. 1c). The resulting optofluidic PAD is composed of three main zones: sample loading, MDF gate for flow control, and PhC film for colorimetric detection (Fig. 1d).

**Fig. 2:**
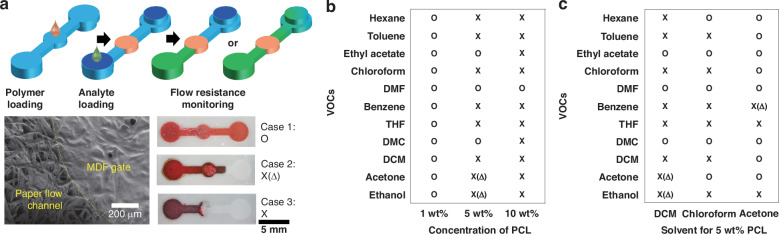
Characterization and optimization of a MDF gate for distinguishing chemical substances. **a** Schematic of fluid flow coding of the chemical analyte using MDF gates. The scanning electron microscopy image shows the boundary between a MDF gate with polymer-embedded and flow channels with neat paper. Optical images show three different cases of fluid flow control using a MDF gate: Pass (case 1, O), Intermediate (case 2, X(Δ)), and Stop (case 3, X). **b**, **c** The evaluation of the flow stop-or-go using 11 organic chemicals at various polymer loading for PCL MDF gates. **b** Effect of PCL concentration on the flow stop-or-go through the MDF gates. **c** Effect of 5 wt% PCL on the flow stop-or-go through the MDF gates

Designing a MDF gate for fluid manipulation requires spatial flow-resistance control over the interaction between the analytes and the polymer to define the flow stop-or-go. To achieve the flow-dependent discrimination of chemical substances in the optofluidic PAD, we introduce MDF gates for passive liquid flow control by loading polymers into the flow control zone of the microfluidic paper (Fig. 2a, the top of the schematic illustration). The five MDF gates are made of polydimethylsiloxane (PDMS), polyvinyl alcohol (PVA), polycaprolactone (PCL), poly(lactic-co-glycolic acid) (PLGA), and polyvinylpyrrolidone (PVP), respectively. The SEM image shows the polymer embedded in the cellulose fibril paper network, referred to as the MDF gate (Fig. 2a, bottom left). The fluid flow dynamics (stop or go) depends on the combination of the MDF gates and chemical analytes and can be monitored by adding dyes directly into the chemical solution. According to the types of organic chemical, we find that MDF gates determine the flow patterns in three different ways (Fig. 2b, bottom right). In the first case, a chemical substance passes directly through the MDF gate and reaches the detection zone (case 1, pass). In the second case, a chemical substance passes through the MDF gate slowly and ultimately does not reach the detection zone (case 2, intermediate pass). In the third case, a chemical substance neither passes the MDF gate nor reaches the detection zone (case 3, stop). Based on the observation, we proceed with further analysis by defining the two states of a MDF gate as the pass gate (O) and the stop gate (X), based on whether a chemical substance reaches the detection zone.

A MDF gate needs to control the fluid flow depending on the target chemical, which can be easily tuned by embedding the appropriate polymer in the paper fibril network. To examine the chemical substance discrimination ability by polymer loading conditions for MDF gate formation, we select PCL as a model polymer, prepare different MDF gates by varying concentrations of PCL and organic solvents, and perform a flow stop-or-go test for 11 organic chemicals. As the concentration of PCL in dichloromethane (DCM) increases, the MDF gate becomes a stop gate for more chemical substances, as shown in Fig. 2b. The MDF gate made of 1 wt% PCL loses its solvent discrimination capability due to the poor filling of the cellulose fibril network, thus becoming a pass gate for all chemical substances. Conversely, the MDF gate made of 10 wt% PCL becomes a stop gate for all chemical substances except dimethylformamide (DMF). In addition to polymer concentration, when chloroform and acetone are used as solvents for a PCL MDF gate, the flow patterns of chemical substances slightly change (Fig. 2c). It is noteworthy that DCM did not pass the PCL MDF gate despite being a good solvent for PCL. Therefore, the MDF gate polymer’s solubility in the chemical substance is not the only factor determining the flow stop-or-go. To confirm this, we perform a simple flow test in the microfluidic paper with no MDF gate using a PCL solution in DCM. As shown in Fig. S2a, pure DCM flows well through the paper channel, showing wettability to the hydrophilic channel of the microfluidic paper composed of PFPE channel wall and cellulose fibril paper channel. When we load a 5 wt% PCL solution in DCM, no flow is observed. This implies that PCL may also change the wettability of DCM to the paper channel, proving the complex interaction between the materials: cellulose hydrophilic channel, PFPE hydrophobic barrier, DCM solvent, and PCL. To further validate the effect of the solvent on MDF gate formation, we characterize the surface morphology of the paper after PCL deposition (Fig. S2b). The fibril network is densely filled with PCL when using DCM and chloroform solvents compared with acetone. In addition, we measure the contact angle of the polymer-loading solution onto the surface of the PFPE film (Fig. S2c). The contact angles of 5 wt% PCL in DCM, chloroform, and acetone are measured as 71.4°, 53.2°, and 42.29°, respectively. Although the result does not represent the complicated interaction determining the flow stop-or-go, a significant correlation is expected between the contact angle and the spreading of chemical substances into the patterned PFPE paper channel of the microfluidic paper. Thus, it can be understood that complex interactions between materials can alter the polymer morphology in the fibril network, affecting the flow stop-or-go in the MDF gate. We also test the stop-or-go of MDF gate by repeating the same experiment using 5 wt% PCL loaded microfluidic paper. As a result, there is no significant difference in flow pattern after the 5 repeated experiments, indicating sufficient reliability of the optofluidic PAD (Fig. S2d). In addition, the different rates of analyte flow and color changes can be affected by temperature and humidity because they are based on different interactions between the materials: cellulose hydrophilic channel, PFPE hydrophobic barrier, chemical analyte, and polymer-embedded. Although there are no significant differences under our conditions, which include a temperature range of 15–25 °C and humidity between 30–60%, further investigations are needed for a more detailed optimization of monitoring time.

**Fig. 3:**
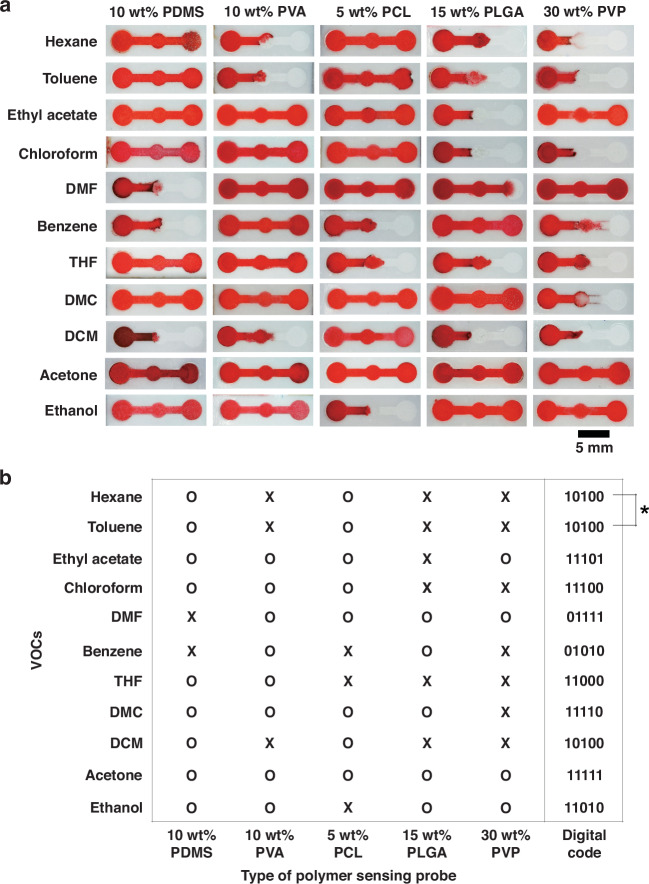
Evaluation of the flow stop-or-go at five different MDF gates. **a** Differential images of the flow stop-or-go under exposure to 11 organic chemicals using PDMS, PVA, PCL, PLGA, and PVP MDF gates. **b** Diagram of flow stop-or-go results (X = stop, O = go), and their conversion to digital codes of “0” and “1”, respectively

The introduction of multiple MDF gates enables precise discrimination of chemical substances in the optofluidic PAD. For screening 11 organic chemicals, five types of MDF gates made of PDMS, PVA, PCL, PLGA, and PVP are tested on the microfluidic paper. They are formed by 10 wt% PDMS, 10 wt% PVA, 5 wt% PCL, 15 wt% PLGA, and 30 wt% PVP solutions in DCM for the best chemical discrimination performance. To visualize the flow pattern, we add red dyes to the chemical solutions. The smallest loading volume of analyte is determined to be 10 μl, at which 11 organic chemicals could be discriminated in our optofluidic PAD capable of minimum reagent consumption. Figure 3a shows photographs of the distinctive stop-or-go flow patterns by chemical substances on the microfluidic paper with different MDF gates, providing molecular fingerprint information for digital encoding and decoding. Figure 3b presents the graphic information on the “O” (pass) and “X” (stop) gates converted to digital codes: “1” for pass gate (O) and “0” for stop gate (X). Each chemical substance exhibits a different digital code with a few exceptions for hexane and toluene (indicated “*”). Despite using five MDF gate types, some chemical substances with similar physicochemical properties are still difficult to distinguish. Therefore, the method requires an additional step for precise discrimination of those as well.

**Fig. 4:**
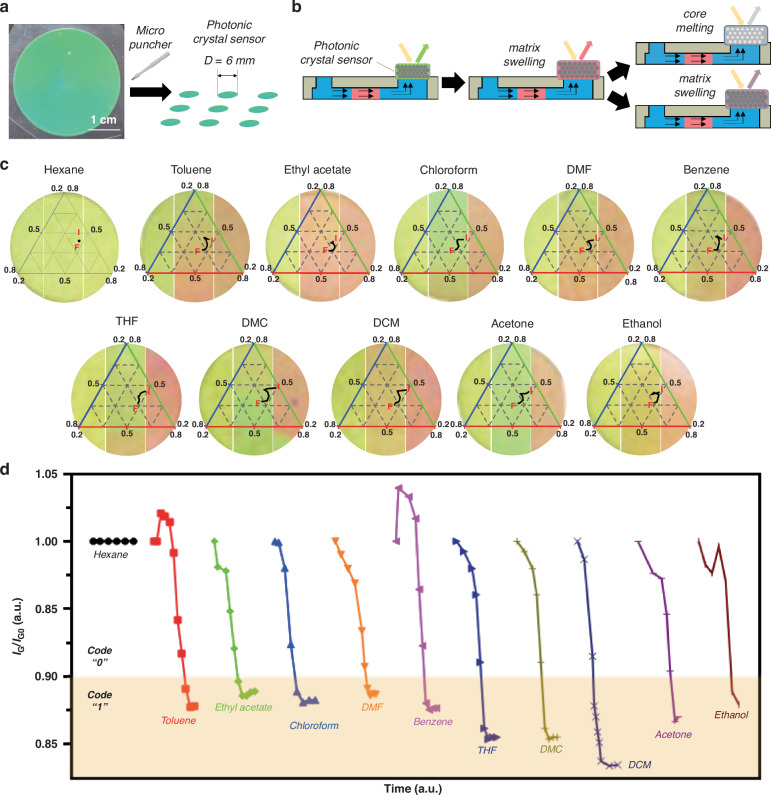
Characterization of colorimetric change in PhC film for different chemical substances. **a** Digital photo image of PhC film made of core-shell nanoparticles (left) and schematics for the preparation of the PhC sensors (right). **b** Schematics for the solvatochromic coding on the PhC-integrated microfluidic paper. **c** Change in reflection color of PhC sensors exposed to various chemical substances represented in the Maxwell color triangle. The color information is traced from the initial “I” state to the final “F” state. The set of optical microscope images in the background for each chemical substance represents the initial (left panel), intermediate (middle panel), and final (right panel) state of PhC sensors, respectively. **d** Change in normalized green color intensity (*I*_*G*_*/I*_*G0*_) of PhC sensors exposed to various chemical substances. Based on the normalized green color intensity, the signals are defined as “1” (colored box) for values below 0.9 and “0” otherwise

Naked-eye detection of chemical substances on the optofluidic PAD can be implemented by integrating colorimetric coding units at the detection zone. We use a colloidal PhC film showing a green reflection color, which is prepared by the melt-shear assembly of polystyrene core/poly(ethyl acrylate) (PEA) shell nanoparticles (Fig. S3a). The SEM image shows a hexagonal array of nanoparticles, which indicates the formation of face-centered cubic lattices (Fig. S3b). The polystyrene core forms a non-close-packed crystal structure surrounded by PEA, as the PEA shell is fused to form a monolithic matrix during the melt-shear assembly process. In a PhC film with such a crystal structure, the structural color is determined according to Bragg’s diffraction equation for (111) planes:

1 $${\rm{\lambda }}=2{{dn}}_{{eff}}={\left(\frac{\pi }{3\sqrt{2{\rm{\phi }}}}\right)}^{\frac{1}{3}}{\left(\frac{8}{3}\right)}^{\frac{1}{2}}{D}_{p}{\left({n}_{p}^{2}{\rm{\phi }}+{n}_{m}^{2}\left(1-{\rm{\phi }}\right)\right)}^{\frac{1}{2}},$$

where *d* is the lattice spacing, *n*_*eff*_ is the effective refractive index, *D*_*p*_ is the particle diameter, *n*_*p*_ and *n*_*m*_ are the refractive indices of the particles and matrix, respectively, and ϕ represents the volume fraction of the particles. Since PEA has high solvent absorption characteristics, it enables a notable and irreversible reflection color change of the PhC film, as the swelling of PEA changes its crystal lattice as per Eq. 1. Although changes in the effective refractive index of the matrix due to the absorption of organic chemicals can also cause changes in reflection color, the magnitude of the refractive index change due to the absorption of organic solvents is not significant because the PhC film is composed of a polymer matrix. Therefore, we focus on the effect of the lattice spacing changes by swelling of PhC film. The PhC film is cut into circles 6 mm in diameter using a micropunch to integrate on the microfluidic paper (Fig. 4a). As shown in Fig. 4b, when an organic chemical passes through the MDF gate and arrives at the PhC film, the PhC polymer matrix expands, causing a red shift of reflection color. Ultimately, PhC either melts or expands, further losing its unique reflection color. For a detailed investigation of the colorimetric behaviors of the PhC in the microfluidic paper, we take video and obtain time-lapsed photos of the PhC film exposed to various chemical substances (Video S1 and Fig. S4). All 11 organic chemicals (introduced at 10 µL each) reach the detection zone within 30 s. Subsequently, the film’s reflection color begins to bleach within tens of seconds for most chemical substances. In contrast, for ethanol and hexane, the reflection color slowly changes and barely change, respectively, because the affinity of ethanol and hexane for PEA is notably lower than that of other organic chemicals. To obtain quantitative results from the time-lapsed images, we track the reflection color change of the PhC film for the 11 organic chemicals. As shown in Fig. 4c, the color information of the PhC film is analyzed using the Maxwell color triangle. All samples show a notable decrease in green color fraction and only subtle changes in red and blue color fractions. Therefore, we establish quantification criteria for color changes caused by various VOCs based on the green color information (see details in Supporting Information). From the optical microscope images, the green color signal is selectively chosen to make grayscale images and we monitor changes in the green color intensity of the PhC film upon exposure to various chemical substances (Fig. S5). The color change is visually detectable when the green color signal falls below a certain level, regardless of the PhC response time (Fig. S5). As shown in Fig. 4, a decrease in intensity is observed for all solvents except hexane, resulting in the normalized signal being reduced below 0.9 of the initial signal. Such behavior enables naked eye detection of any unknown solvent passing through the MDF gate. To quantify its presence based on the green color signal change, we assign the initial signal of the PhC film the code “0”, which should change to “1” if the normalized intensity is below 0.9.

**Fig. 5:**
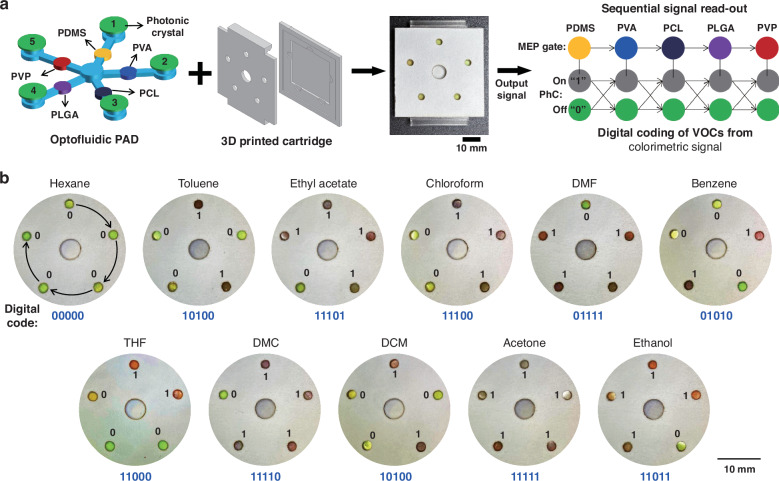
Optofluidic digital coding of chemical substances. **a** Schematic of an optofluidic PAD and the signal readout procedure. The microfluidic paper with five MDF gate channels is assembled with the PhC film and a 3D-printed cartridge. The photo image shows the resulting optofluidic PAD. After loading the liquid analyte, colorimetric signals are sequentially interpreted, starting from PDMS to PVP, and converted into digital codes. **b** Differential photo images of the optofluidic PAD after loading 11 different organic chemicals, indicating digital codes for each chemical substance

We combine multiple MDF gates and PhC films in the optofluidic PAD and test their discrimination capability for 11 representative chemical substances. We also design a bubble-map-array microfluidic paper to integrate five MDF gates and PhC film into it (Fig. 5a). Five MDF gates comprising PDMS, PVA, PCL, PLGA, and PVP form the flow control zone of the microfluidic paper, with PhC films attach to each detection zone. The PAD is integrated into a 3D-printed cartridge for easy use and minimal evaporation. To test the discrimination of these chemical substances, we load 50 μL of each sample to the analyte-loading zone at the chip center and wait for 2 min to acquire the colorimetric result. The differential color bubble-maps, displayed as digital codes are shown in Fig. 5. These optofluidic digital codes form the instructions needed to identify the VOCs. All 11 organic chemicals have different codes and are thus successfully discriminated using our optofluidic PAD. Notably, hexane and toluene show different results in the detection zone due to their different interactions with the PhC film. This proves that the PhC film further screens the solvents with identical flow patterns after passing through the polymer gateway. Thus, further development of MDF gates and PhC materials will allow for more diverse discrimination of materials in the future.

## Conclusion

The primary aim of the study is to demonstrate the feasibility of discriminating various chemical substances using an optofluidic PAD comprising a microfluidic paper with integrated MDF gates and PhC films. The microfluidic paper with a hemi open channel is fabricated by double-sided photolithography using an organic chemical compatible PFPE-DMA. By combining fluidic and optical phenomena based on MDF gates and PhC films, respectively, we successfully convert the information on chemical molecular fingerprints into simple digital codes by reading colorimetric signals from a bubble-map array on the optofluidic PAD. The unique optofluidic digital codes for various chemical substances are demonstrated through the complex physical and chemical correlation between chemical molecules and materials constituting the optofluidic PAD, including the cellulose network, PFPE hydrophobic barrier, MDF gates, and PhC film. The discrimination performance can be further improved by integrating various coding probes and detectors. For example, an optofluidic PAD with over five integrated MDF gates using various polymers or an integrated smartphone for quantifying colorimetric changes of PhC film will allow us to distinguish a wider variety of chemical substances and complex mixtures more effectively. In more detail, the structural colors of PhC film can be changed dynamically during the absorption and drying of organic chemicals, which may also allow for more sophisticated discrimination of analytes^25,26^. In addition, integration of additional probes with different detection mechanisms, such as a molecular-imprinted polymer and surface plasmon resonance-mediated colorimetric sensors, with our optofluidic PAD could also enhance the detection limits for various chemical types and mixtures. Therefore, we believe that our strategy provides a facile and easy-to-perform assay platform for coding various chemical substances, presumably paving the way to scalability by integrating multiple coding approaches for broad application to colorimetric monitoring of chemicals observed in environment.

## Experimental section

### Fabrication and characterization of the monolithic microfluidic paper

The photocurable PFPE-DMA (Fluorolink MD 700, Solvay, Belgium) is used to form a solvent-resistant hydrophobic barrier via UV irradiation. The PFPE-DMA solution is prepared by adding 2-hydroxy-2-methylpropiophenone (Darocur 1173, Sigma-Aldrich, MO, USA) at 5 wt% as the photoinitiator. Figure 1b shows the microfluidic paper fabrication procedure via double-sided patterning. Whatman cellulose chromatography paper (Grade 3 MM Chr, Sigma-Aldrich) is cut into pieces by a laser machine (Nova 24, Thunder Laser, China) and soaked in 50 mL of the PFPE-DMA solution for 60 min. Excess PFPE-DMA solution is removed from the paper, and then the soaked paper is placed between two photomasks of different designs, aligned and fixed by double-sided tape. The top side of the paper is exposed to 350-nm UV light (24 mW/cm^2^) for 5 s. After that, the bottom of the paper is exposed to UV light for 5 s under the same conditions. The uncured PFPE-DMA solution is removed by sonicating the paper immersed in diethyl ether (Sigma-Aldrich) for 2 h, and the paper is dried at room temperature for 1 day. To prepare a MDF gate for the microfluidic paper, 1.5 μL of a polymer solution is loaded on the MDF gate zone and dried at room temperature for 24 h.

### Preparation of the PhC film

For the fabrication of PhC film, we conduct the melt-shear assembly of core-shell nanoparticles: polystyrene core, poly(methyl methacrylate) interlayer, and poly(ethyl acrylate) shell synthesize using starved-feed emulsion polymerization^27,28^. For the bright structural color of the colloidal PhC film, polydopamine is incorporated into the core-shell nanoparticle dispersion by oxidative polymerization. The details will be published elsewhere. Before the melt-shear assembly, the particles were centrifuged at 8000 rpm for 30 min and dried in a convection oven at 80 °C for a day after removing the supernatant. Subsequently, 0.1 g of the dried particles are placed between polyethylene terephthalate(PET) films (thickness = 80 μm) and pressed at 110 °C using a hot press machine (QM900M, QMESYS, South Korea) at 10 MPa to form the PhC film. To prepare the coding units, the PET film is removed, and the PhC film was cut into circles 6 mm in diameter using a micropunch.

### Fabrication of the 3D-printed cartridges

First, to integrate the PhC film into the microfluidic paper, we design two cartridges using 3D computer-aided design software (Inventor 2022, Autodesk, CA, USA). The top cartridge has a negative pattern for fixing PhC film and holes for sample loading and detection. The bottom cartridge has a negative pattern to prevent the cartridge surface from wetting by the analyte of the paper channel. The cartridges are printed using a PolyJet 3D printer (Objet30 Prime, Stratasys, MN, USA) with a Veroclear transparent resin as the base material and SUP706 as the support material. The resolution of the 3D printer is: *x*-axis 600 dpi, *y*-axis 600 dpi, *z*-axis 1600 dpi, and 16 μm layer thickness. The printed cartridges are immersed in a washing solution comprising 2% sodium metasilicate anhydrous (Na_2_SiO_3_, Duksan, South Korea) and 1% sodium hydroxide (NaOH, Duksan) in deionized (DI) water and sonicated for 2 h to remove the support material.

### Preparation and operation of the optofluidic PAD for digital coding of chemical fingerprint

The optofluidic PAD is prepared by integrating the microfluidic paper and PhC film into 3D-printed cartridges, as shown in Fig. 1c. For the first coding unit, we select five polymers with solvents (10 wt% PDMS in hexane, 10 wt% PVA in DI water, 5 wt% PCL in acetone, 15 wt% PLGA in acetone, and 30 wt% PVP in DI water). These chemicals are purchased from Sigma-Aldrich. Each polymer solution (1.5 μL) is dropped on the flow control zone of the microfluidic paper and dried at room temperature for 1 day. We prepare a 6 mm diameter PhC film covered with a PET layer that can protect the crystal structure from varying external conditions (humidity, dust, and pollutants), and then place it onto the detection zone of the microfluidic paper with the PhC layer facing up. Finally, the microfluidic paper is assembled with a 3D-printed cartridge, as shown in Fig. 1d. This cartridge can also protect the microfluidic paper from fluctuating external conditions. For optofluidic coding of chemical substances, 50 μL of analyte sample is loaded into the loading zone of the optofluidic PAD, and colorimetric changes are identified in the detection zone after 2 min under normal conditions with a temperature range of 15 ~ 25 °C and humidity range of 30 ~ 60%.

### Characterization

The PFPE pattern formation is characterized by adding 1 wt% methylene blue dye in DI water to the paper channel. The solvent resistance and the fluid flow of the microfluidic paper are characterized by adding 1 wt% Oil red O dissolved in each chemical substance. The morphologies of the polymer-embedded paper are characterized by SEM (Sigma 500 VP, Zeiss, Germany). All optical images are obtained by using a stereo microscope (Eclipse Ti2-U, Nikon Instruments, Japan) with a digital camera (DS-Qi2, Nikon Instruments) supported by NIS-Elements AR software and analyze with ImageJ (National Institutes of Health, MD, USA).

## Supplementary information

Supplementary Information

Video S1. Colorimetric change of PhC for chloroform analyte
