# Supplementary material for: Optofluidic paper-based analytical device for discriminative detection of organic substances via digital color coding
Source: Microsyst Nanoeng. 2025 Jan 16;11:11. doi: 10.1038/s41378-024-00865-4 (PMC11739424; doi:10.1038/s41378-024-00865-4)
Supplement: Supplementary file 1 — Supplementary Information [file 41378_2024_865_MOESM1_ESM.docx]

**SUPPLEMENTARY INFORMATION**

**Optofluidic paper-based analytical devices for digital coding of chemical fingerprints for discriminative detection**

Jinsol Choi,^a,†^ Chi Yeung Oh,^b,†^ Gong Qian,^a^ Tae Soup Shim,^b,c,^* and Heon-Ho Jeong^a,^*


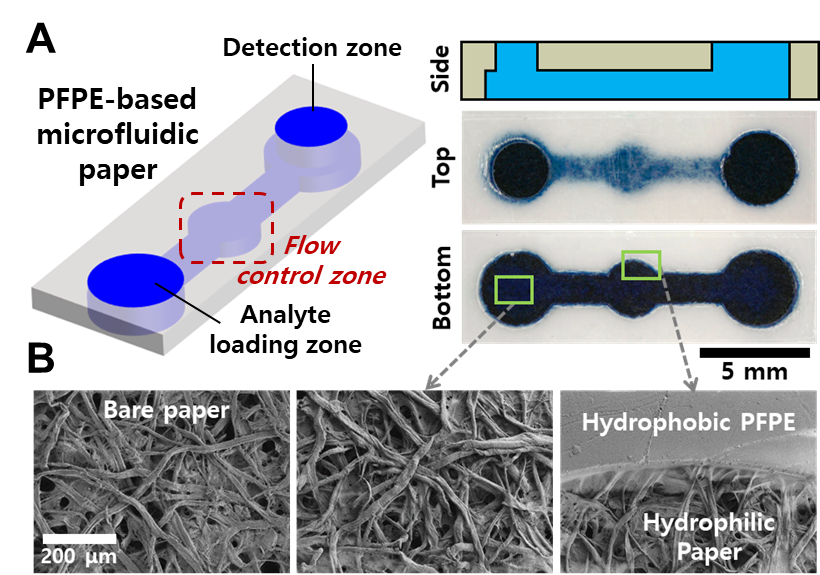


**Figure S1.** 3D-patterned PFPE barrier within the monolithic microfluidic paper. (A) Schematic and optical images of the 3D patterned PFPE barrier on the top and bottom of the paper. (B) SEM images for bare paper (left image) and two regions of fabricated microfluidic paper: hydrophilic paper channel (middle image) and hydrophobic PFPE barrier (right image).


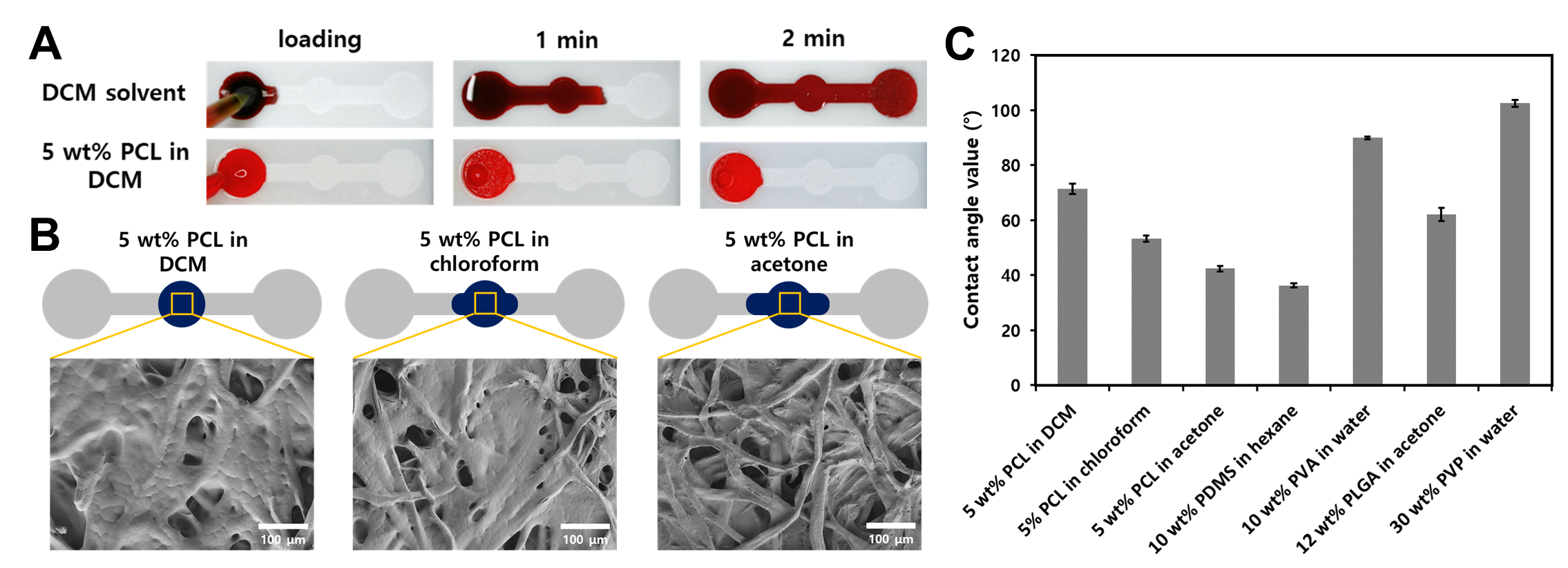


**Figure S2.** Characterization of MDF gate formation. (A) Fluid flow test on the hemi channel of microfluidic paper using DCM and 5 wt% PCL in DCM solution. Optical images show time-lapsed fluid flows for 2 min. (B) SEM images for the formed PCL MDF gate for various solvents. (C) Contact angle of solution condition for MDF gate formation onto the PFPE thin film


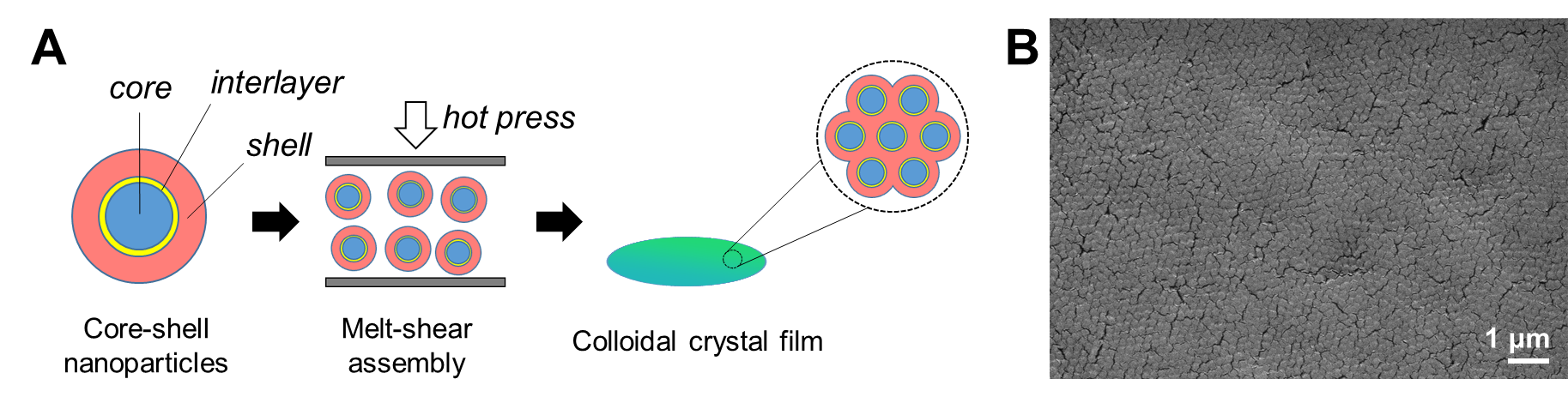


**Figure S3.** Fabrication of PhC film. (A) Schematics for the fabrication of the colloidal PhC film by the melt-shear assembly of core-shell nanoparticles. (B) SEM images of the colloidal photonic crystal film after the melt-shear assembly of polystyrene core/poly(ethyl acrylate) shell nanoparticles


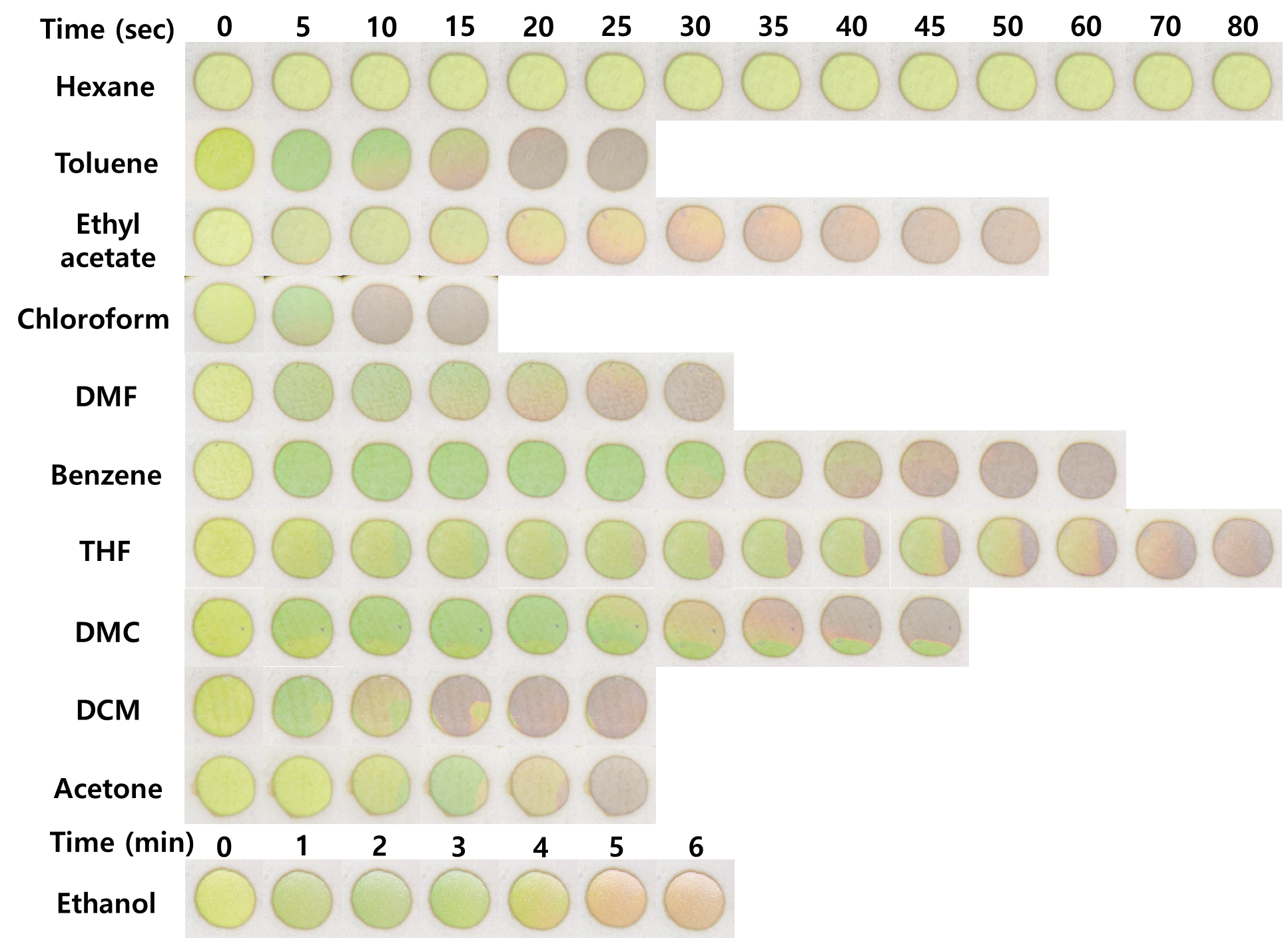


**Figure S4.** Time-lapsed photo images of change in structural color of PhC films for 11 organic chemicals.


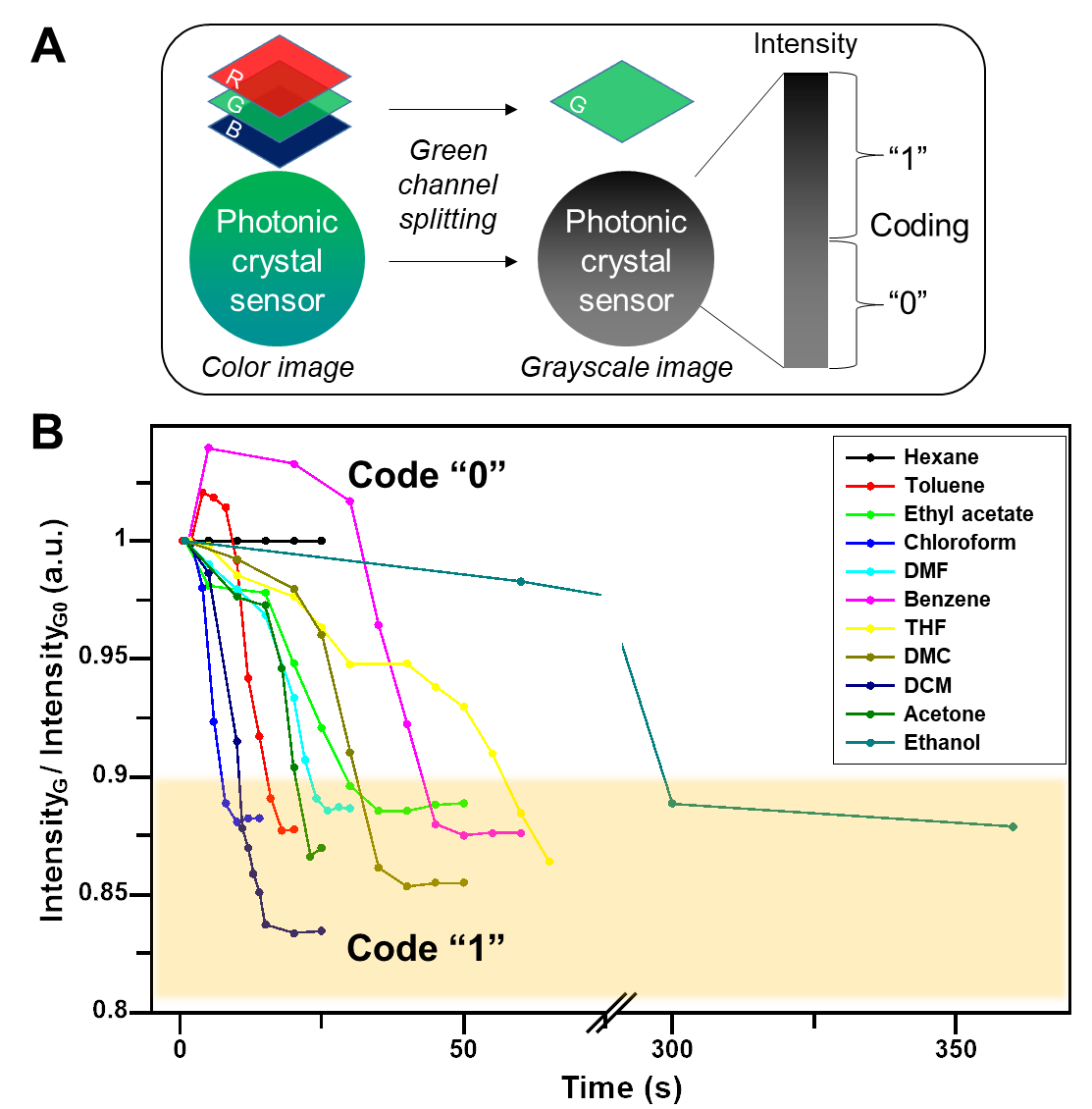


**Figure S5.** Characterization of colorimetric change in the PhC film for different chemical substances (A) Illustration of the procedure for separating, gray-scaling, and rescaling green color information from RGB color images for qualitative analysis of the optical microscopy images. (B) Graph showing the change in normalized light intensity of green color (*I_G_/I_G0_*) over time for 11 organic chemicals.
